# Re-Treatment with EGFR-TKIs in NSCLC Patients Who Developed Acquired Resistance

**DOI:** 10.3390/jpm4030297

**Published:** 2014-06-25

**Authors:** Wen-Shuo Wu, Yuh-Min Chen

**Affiliations:** Department of Chest Medicine, Taipei Veteran General Hospital, Yang-Min National University, No. 21, Sec. 2, Shi-Pai Rd., Taipei 11217, Taiwan; E-Mail: marco1215@gmail.com

**Keywords:** epidermal growth factor receptor (EGFR), tyrosine kinase inhibitor (TKI), non-small cell lung cancer (NSCLC)

## Abstract

In the era of personalized medicine, epidermal growth factor receptor (EGFR) inhibition with tyrosine kinase inhibitor (TKI) has been a mainstay of treatment for non-small cell lung cancer (NSCLC) patients with an *EGFR* mutation. Acquired resistance, especially substitution of methionine for threonine at position 790 (T790M), which has accounted for more than half of the cases, developed inevitably in patients who were previously treated with EGFR-TKI. At present, there is no standard treatment for patients who have developed a resistance to EGFR-TKI. Several strategies have been developed or suggested to treat such patients. This article aimsto review the EGFR-TKI re-treatment strategy and the efficacy of different generations of EGFR-TKIs in patients with acquired resistance to prior EGFR-TKI.

## 1. Introduction

Lung cancer is the leading cause of global cancer death. Patients are diagnosed mostly with non-small cell lung cancer (NSCLC) and usually have a poor prognosis [1,2]. The Iressa-Pan-Asia study, which is the first study to demonstrate that patients with special characteristics, female, non-smoker and adenocarcinoma, have a better response to gefitinib than conventional platinum-based doublet chemotherapy [3,4,5], in terms of response rate (RR), progression-free survival (PFS) and quality-of-life. The discovery of a mutation on the epidermal growth factor receptor (*EGFR*) gene made a huge difference in the treatment strategy for NSCLC, especially adenocarcinoma. The *EGFR* exon 19 deletion, or exon 21 L858R point mutation, is predictive of a treatment advantage with EGFR-tyrosine kinase inhibitor (EGFR-TKI) therapy [6,7]. The presence of an activating *EGFR* mutation in the tumor varies among different races [8]. About 10% to 15% of patients harboring an activating *EGFR* mutation are from Western societies [9], and about 50% are Asian [10,11]. The findings of the high efficacy of EGFR-TKI treatment in patients with activating *EGFR* mutations have had a great influence on the paradigm of NSCLC treatment. Since two Asian studies demonstrated a more than 70% RR in the activating *EGFR* mutation subgroup [5,12], several randomized prospective trials have confirmed that first-line EGFR-TKI in patients with activating *EGFR* mutations significantly improved the RR and PFS compared with standard platinum-based chemotherapy [13,14,15]. However, about 60% of patients with disease progression after the initial response to EGFR-TKI are associated with acquired resistance to EGFR-TKI, the substitution of methionine for threonine at position 790 (T790M) point mutation [16,17,18]. There is currently no standard treatment for patients resistant to EGFR-TKI. In this article, we want to review re-treatment with EGFR-TKI in NSCLC patients with activating *EGFR* mutation.

## 2. Acquired Resistance to EGFR-TKIs

Patients with an activating *EGFR* mutation developed resistance eventually, with a median PFS of approximately 8–11 months, when treated with gefitinib or erlotinib [5,12,15,19,20]. Jackman and colleagues [21] suggested a basic definition for acquired resistance to previous use of EGFR-TKI in order to provide a more uniform approach to investigation in further studies. Various mechanisms were later identified, and it is crucial to understand them so as to develop a strategy to overcome resistance. The *EGFR* exon 20 T790M point mutation is the first and most frequently reported mechanism [17,22,23,24]. It was initially proposed based on the crystallographic structure of the *EGFR* tyrosine kinase domain. The bulkier methionine residue T790M changes the ATP-binding pocket of the tyrosine kinase domain, leading to the blockade of gefitinib or erlotinib [22]. However, it was demonstrated recently that T790M increased the affinity of ATP to the *EGFR* tyrosine kinase domain; thus, it decreased the binding of gefitinib and erlotinib, because they are ATP-competitive agents [25]. Other acquired resistance mechanisms, such as small cell transformation, *MET* amplification, epithelial-mesenchymal transition and *PIK3CA* mutation, were found in small series of patients [17,24,26,27]. A small study in Korea demonstrated a similar result to the Western population [24]. The presence of T790M defines a clinical subgroup with a more favorable prognosis and more indolent progression among patients with acquired resistance [28,29]. Still, one-third of the mechanisms of acquired resistance are not yet understood [30].

In clinical practice, we usually define patients who develop resistance to EGFR-TKI treatment into three groups: those with oligo-site progression, systemic and multiple progression and isolated central nervous progression [31]. For those patients with oligo-site progression or isolated CNS progression, local treatment to the progression site and continuation of the present EGFR-TKI treatment is suggested [32,33,34]. For patients with a slowly progressing lesion(s) and with lesions smaller than pre-treatment and progression, as documented by Response Evaluation Criteria In Solid Tumor (RECIST), and without the worsening of systemic symptoms and/or signs, the continuation of the present EGFR-TKI is also suggested [20,35]. Thus, the present review of EGFR-TKI retreatment is focused mainly on those patients who need new systemic treatment and excludes those with slowly progressing tumor, oligometastases or isolated CNS metastases. In addition, only those modalities that involve re-treating with EGFR-TKI are discussed.

## 3. Continue or Re-Treat with First-Generation EGFR-TKIs

Several first-generation EGFR-TKI treatment strategies have been developed for patients with an activating *EGFR* mutation after disease progression. Second-line chemotherapy is a reasonable choice, despite the lack of prospective evidence for this subset of patients. The subgroup analysis of the TORCH trial [35,36] demonstrated a 15% RR and a four-month median PFS in patients with an *EGFR* mutation who received chemotherapy after erlotinib. Discontinuation of EGFR-TKI may lead to rapid tumor regrowth [37,38] and many patients developed asymptomatic or slowly progressing disease. Thus, some oncologists chose to continue TKI and delayed further salvage chemotherapy [28]. Some retrospective studies supported the continuation of gefitinib in those who initially responded to gefitinib [39,40]. ASPIRATION (NCT01310026) is a large, prospective, multi-center, single-arm trial to evaluate the efficacy of first-line erlotinib in NSCLC patients harboring an *EGFR* mutation beyond disease progression [41]. The trial is ongoing and may give us more information about the continuation of EGFR-TKI in patients beyond progression.

The addition of chemotherapy and the continuation of EGFR-TKI have also been considered. A retrospective study [42] was performed with 16 NSCLC patients who had partial or complete response to prior gefitinib. They received continued gefitinib plus paclitaxel after disease progression. The study showed a 13% objective RR, a median PFS of 4.3 months and an overall survival (OS) of 8.1 months. Another retrospective study [43] reported a similar result. Seventy-eight advanced NSCLC patients with an EGFR mutation and acquired resistance received subsequent chemotherapy plus erlotinib or chemotherapy alone. The objective RR of 57 evaluable patients was 41% for those who received chemotherapy plus erlotinib and 18% for those who received chemotherapy alone (HR = 0.31, 95% CI = 0.09–1.04; *p* = 0.08). The median PFS of patients that received chemotherapy plus erlotinib was 4.4 months and for those that received chemotherapy alone, 4.2 months (*p* = 0.34). Worthy of noting is that 94% of patients in the chemotherapy plus erlotinib group had erlotinib as the initial TKI. There was no difference in OS between these two groups. In addition, a small prospective study was performed to evaluate patients with the *EGFR* mutation who were under treatment with gefitinib or erlotinib. It showed a 25.9% of overall RR and 77.8% disease control rate in those who were treated with gefitinib or erlotinib in addition to pemetrexed after disease progression [44]. There is an ongoing clinical trial (IMPRESS, NCT01544179) comparing the continuation of gefitinib with the addition of cisplatin plus pemetrexed *versus* cisplatin plus pemetrexed in NSCLC patients who have progressed on first-line gefitinib.

The other strategy is to re-treat with EGFR-TKI after progression with second-line salvage chemotherapy. A pre-clinical model revealed the re-gaining of sensitivity to TKI in NSCLC cell lines [45]. Several small retrospective studies found that patients can still be sensitive to EGFR-TKI after stopping treatment [46,47]. Another retrospective study reported a small group (14 patients) of patients that was heavily treated and then re-treated with erlotinib after a median interval from the discontinuation of EGFR-TKI to the second episode of 9.5 months (3–36 months) [48]. The RR was 36% (*n* = 5), and the disease control rate was 85.7% (*n* = 12). Before beginning the re-treatment, T790M was detected in 36% of patients (five of 14). Two of the five patients had a partial response; one had stable disease, and the other two had progressive disease [48]. In the American Society Clinical Oncology (ASCO) 2012 meeting, a small series of patients with *EGFR* mutation was re-treated with EGFR-TKI, which had a beneficial effect after a drug-free interval (median time: 11 months without TKI). The heavily-treated patients in this group had 4.4 months of PFS [49].

Furthermore, a phase I/II clinical trial with combined cetuximab, *EGFR* monoclonal antibody and erlotinib was conducted based on the hypothesis of overcoming acquired resistance by a combined *EGFR* pathway blockade [50]. However, the adenocarcinoma patients with an *EGFR* mutation who developed acquired resistance to erlotinib failed to show an objective response.

In patients with an *EGFR* mutation or that responded to prior EGFR-TKI either continued TKI combined with chemotherapy or re-treatment with EGFR-TKI after a drug-free interval could be considered as a further treatment strategy.

## 4. Change to Second-Generation EGFR-TKIs

The resistance to first-generation EGFR-TKIs developed inevitably during treatment. The second-generation of EGFR-TKIs were then developed with some theoretical advantages. Compared to the reversible EGFR-TKIs, the irreversible second-generation TKIs have a higher affinity to tyrosine kinase, which may result in a longer blockade of signaling [51]. Besides, the second-generation EGFR-TKIs are pan-HER inhibitors and may block the *EGFR* signaling pathway more completely. They also have *in vitro* activity against the T790M mutation. Of the second generation EGFR-TKIs, neratinib, dacomitinib and afatinib have been getting more attention recently (Table 1).

Neratinib (HKI-272) is an oral, irreversible inhibitor of *EGFR* and HER2 [52]. In pre-clinical studies, neratinib inhibited the growth of NCI-H1975 bronchoalveolar cancer cells harboring both substitution of arginine for leucine at position 858 (L858R) and T790M and cell lines harboring the *HER2* mutation [51]. A phase I study of the advanced stage of solid tumors showed that the maximum tolerated dose (MTD) of neratinib was 320 mg once daily [53]. An open-label, single-agent, phase II study revealed only a 3% RR in patients with an *EGFR* mutation who had been treated with gefitinib or erlotinib for more than 12 weeks. The median PFS was 15.3 weeks in this subset of patients [54]. Of note, three of four patients (75%) with a substitution of amino aicd for glycine at position 719 (G719X) mutation in *EGFR* achieved a partial response. The median PFS was 52.7 weeks for these four patients. Since the diarrhea (46% in grade 3/4) and other adverse events (58% in grade 3/4) were unacceptably high, the dosage in this study was decreased to 240 mg daily, which may not achieve a therapeutic level [54]. Neratinib is not being developed for NSCLC currently, due to the disappointing result.

Dacomitinib (PF-00299804) is an irreversible pan-HER inhibitor that binds to each of the three tyrosine kinase active members of the HER family. In preclinical models, dacomitinib was highly potent in inhibiting the HER domain and anti-cancer activity in gefitinib-resistant cell lines and xenograft with *HER2* and T790M mutation NSCLC models [55,56]. A phase I study was conducted with patients with advanced malignant solid tumors and revealed the MTD of a dacomitinib dose to be 45 mg once daily [57]. In a phase II trial [58], NSCLC patients with one or two prior chemoregimens were included. Dacomitinib was compared with erlotinib as a second or third-line treatment. The median PFS in this study was 2.86 months with dacomitinib and 1.91 months with erlotinib (hazard ratio (HR) = 0.66; 95% CI, 0.47 to 0.91; two-sided *p* = 0.012). The subgroup analysis was more in favor of dacomitinib in the *EGFR* mutation group (hazard ration (HR) = 0.46; 95% CI, 0.18 to 1.18). In another phase II study [59] with 66 patients previously treated with one or two chemotherapy regimens and erlotinib, dacomitinib demonstrated a 5% objective RR in patients with adenocarcinoma. Median PFS was 12 weeks overall and 18 weeks in patients with an *EGFR* mutation. Gastrointestinal and dermatological adverse events, predominantly grade 1 to 2, were more common in the dacomitinib group than with erlotinib [58,59]. Based on the above data, a randomized, double-blinded, phase 3 study (ARCHER 1009; NCT01360554) comparing dacomitinib to erlotinib, was conducted with advanced NSCLC patients with at least one prior treatment. However, it failed to meet its objective of demonstrating statistically significant improvement in PFS when compared to erlotinib. The BR.26 trial, a double-blind, placebo-controlled, randomized study, comparing dacomitinib and placebo in patients with advanced NSCLC after standard therapy with both chemotherapy and an EGFR-TKI, also had failed to meet its objective of prolonging OS *versus* placebo. Another clinical trial (ARCHER 1050; NCT01774721) comparing dacomitinib to gefitinib in advanced NSCLC with *EGFR* mutation in a first-line setting is also ongoing.

**Table 1:** The second generation EGFR-TKIs.

Agent	Phase	*N*	Patient Population	ORR (%)	DCR (%)	PFS (mon)	OS (mon)	Ref.
Neratinib	2	91	*EGFR* mutation, prior treatment with EGFR-TKI	3.4	53.4	3.8	N/A	[50]
	2	48	*EGFR* wild type, prior treatment with EGFR-TKI	0	64	4.0	N/A	[50]
Dacomitinib	2	23	*EGFR* mutation, prior treatment with chemotherapy and/or EGFR-TKI	9	30	4.5	14.8	[55]
	2	94	NSCLC, prior treatment with one or two chemoregimens, EGFR-TKI naive	17	N/A	2.86	9.53	[54]
	2	19	*EGFR* mutation, prior treatment with one or two chemoregimens, EGFR-TKI naïve	N/A	N/A	7.44	N/A	[54]
Afatinib	2	61	Adenocarcinoma, prior treatment with EGFR-TKIs and chemotherapy for ≥12 weeks	8.2	65.6	4.4	19.0	[59]
	2/3	390	Adenocarcinoma, prior treatment with chemotherapy and EGFR-TKIs, EGFR status not reported	7	58	3.3	10.8	[60]

ORR: objective response rate; DCR: disease control rate; PFS: progression-free survival; OS: overall survival.

Afatinib (BIBW 2992) is a highly selective, irreversible inhibitor of *EGFR* and a pan-HER inhibitor [60]. In both *in vitro* and *in vivo* preclinical models, afatinib demonstrated increased affinity to common *EGFR* mutations, as well as the T790M mutation [60]. After several phase I studies [61,62], MTD at 50 mg orally daily was established, and the LUX-Lung series of trials has shown promising results.

First, the single-arm phase II trial (LUX-Lung 4), involving Japanese patients with pulmonary adenocarcinoma that progressed after ≥12 weeks of prior gefitinib and/or erlotinib, has shown a modest effect in a third- or fourth-line setting [63]. The majority of the patients (72.6%) were *EGFR* mutation-positive, and 95.2% of all patients had EGFR-TKI for at least 24 weeks. The median time interval between discontinuation of the previous EGFR-TKI and the start of afatinib was three weeks (ranging from two to 13 weeks); 83.9% of the patients (52 of 62 patients) had an interval of less than four weeks. Fifty-two patients had one chemotherapy regimen and 10 patients had two before starting afatinib. The objective RR was 8.2% (five of 61 evaluable patients, 95% CI, 2.7% to 18.1%), and the disease control rate was 65.6% (40 of 61 evaluable patients, 95% CI, 52.3% to 77.3%). The median PFS was 4.4 months (95% CI, 2.8 to 4.6 months). The predominant adverse events were diarrhea (100%) and rash/acne (91.9%) in all grades.

Second, the phase IIb/III randomized trial (LUX-lung 1) examined the use of afatinib plus best supportive care (BSC) *versus* placebo plus BSC in 585 NSCLC patients who had disease progression with one or two prior chemotherapy regimens and gefitinib/erlotinib [64]. The objective response was 7% (29 of 585 patients) in the afatinib group. The median PFS was 3.3 months (95% CI, 2.79–4.40) in the afatinib group and 1.1 months in the placebo group (95% CI, 0.95–1.68), with an HR of 0.38 (95% CI, 0.31–0.48; *p* < 0.0001). The subgroup analysis was more in favor of afatinib use in *EGFR* mutation-positive patients, with an HR of 0.51 (95% CI, 0.31–0.85). However, the study did not meet its primary endpoint, as the median OS was 10.8 months *versus* 12.0 months (HR = 1.08; 95% CI: 0.86–1.35) for afatinib and the placebo, respectively. Diarrhea (87%) and rash/acne (78%) were the most frequently reported adverse events. Afatinib was further evaluated combined with chemotherapy, like gefitinib, in a multicenter, phase III, randomized study (LUX-lung 5). LUX-lung 5 (NCT01085136) is going to compare afatinib plus paclitaxel *versus* chemotherapy of the investigator’s choice alone following afatinib monotherapy in NSCLC patients failing previous gefitinib or erlotinib treatment. Preliminary results will be reported in the ASCO 2014 annual meeting. Afatinib was also compared with standard chemotherapy in the first-line setting [65,66].

Third, there have been recent advances in the combination of afatinib and cetuximab. In the xenograft model of mice, the combination of cetuximab and afatinib can lead to a significant reduction of erlotinib-resistant tumor harboring T790M, compared to gefitinib plus cetuximab [67]. A phase II study supported by the above preclinical data was then conducted using the combination of cetuximab and afatinib in NSCLC patients who developed acquired resistance to first-generation EGFR-TKIs by clinical definition [68,69]. There were confirmed partial responses reported in eight of 22 patients (36%, 95% CI: 0.17–0.59), including 4/13 patients (29%) with a positive T790M mutation. The median PFS was 4.7 months.

## 5. Third-Generation EGFR-TKIs

The second-generation EGFR-TKIs, such as dacomitinib and afatinib, have shown promising effects in pre-clinical models, but limited activity in clinical trials for patients with acquired resistance to previous EGFR-TKIs, due to their toxicity. A new class of TKIs is under development to target T790M. Among these, CO-1686 and AZD9291 are third-generation EGFR-TKIs currently in an early clinical development status, but with some clinical data available (Table 2).

**Table 2:** Third-generation EGFR-TKIs.

Agents	Number of Patients	Number of Patients with Acquired Resistance and T790M	Objective Response Rate	Ref.
CO-1686	9	n/a	67%	[70]
AZD9291	35	18	43%	[71]

CO-1686, a 2,4-disubstituted pyrimidine compound, is a novel, oral *EGFR* inhibitor for NSCLC. It can inhibit mutant *EGFR in vivo* and *in vitro*, both irreversibly and selectively, particularly in the T790M mutation [70]. In pre-clinical models, it demonstrated anti-tumor activity in human *EGFR*^L858R^- and *EGFR*^L858R/T79^°^M^-expressing transgenic mice. The activity was spared *in vivo* in *EGFR* wild-type receptor signaling [70]. In the 2014 ELCC meeting [72], the result of the phase I part of the phase I/II study of CO-1686 in *EGFR* mutation patients who had disease progression after prior EGFR-TKI was reported. A total of 62 patients were enrolled with doses ranging from 150 mg QD to 900 mg BID in free base formulation and 500 mg QD to 1,000 mg QD in hydrobromide (HBr) salt formulation. About seventy-five percent of them came directly off of TKI with progressive TKI resistance. CO-1686 is well tolerated, and there has only been one patient discontinued due to an adverse event. Treatment-related adverse events were noticed in more than 20% of patients. Grade 3 (19%) hyperglycemia was reported, but it was typically asymptomatic. QTc prolongation grade 3 was observed in 5% of cases and resolved mostly after the dose reduction. Other adverse effects, such as nausea, diarrhea, decreased appetite and fatigue, were noticed in >10% of patients, but mainly in grade 1. Dose-related wild-type-driven diarrhea and skin rashes have not been seen. Based on the promising results, several clinical trials are planned this year to evaluate the efficacy of CO-1686 in different clinical settings.

AZD9291 is a potent, irreversible and effective inhibitor, both in sensitizing *EGFR* mutations and resistant mutations in cell lines *in vitro*, while sparing *EGFR* wild-type [71]. The drug demonstrated anti-tumor activity in *EGFR*-mutant xenografts at a low dose level [71]. A phase I open-label, multi-center trial has been performed with both Asian and Western patients with advanced NSCLC who had disease progression with prior EGFR-TKI therapy. AZD9291 was well tolerated in the trial with no dose-limiting toxicities at doses of 20, 40, 80 or 160 mg/day. The preliminary data [73] showed that 15 of 35 patients had a confirmed and unconfirmed partial response (43%), including nine of 18 patients with positive T790M. One of them, with negative T790M, also had a partial response.

The successful results with CO-1686 and AZD9291 may have a great influence on the treatment of advanced lung cancer with *EGFR* mutations. They both showed activity in sensitizing *EGFR* mutations, as well as resistant mutations. Further clinical trials with third-generation EGFR-TKIs will demonstrate the anti-tumor activities.

## 6. Conclusions

The standard treatment for patients with acquired resistance to EGFR-TKIs has not yet been defined. Chemotherapy is still an acceptable choice for these patients. Re-treatment with first-generation TKIs after a drug-free interval or the combination with chemotherapy in patients beyond progression may have a modest effect. Second-generation EGFR-TKIs seem to have the same activity as first-generation TKIs in heavily-treated patients, although there were very good responses in a first-line setting compared to chemotherapy [65,66]. Furthermore, afatinib in combination with cetuximab had good activity in patients who developed resistance to erlotinib or gefitinib. However, the adverse effects of second-generation EGFR-TKIs are more severe than those of gefitinib or erlotinib. The third-generation EGFR-TKIs, CO-1686 and AZD9291, demonstrated anti-tumor activity in both sensitizing and resistant *EGFR* mutation tumors in preclinical models. The phase I trials of these two drugs showed promising effects in TKI-resistant patients, with a low rate of adverse effects in preliminary reports. Further trials are ongoing currently.
